# The Effectiveness of a Social Skills Training Intervention Program for Family Members of Patients With Schizophrenia in Japan: A Pilot Study

**DOI:** 10.7759/cureus.109767

**Published:** 2026-05-27

**Authors:** Misuzu Takahara, Tetsuya Miyagi, Daisuke Takahara, Noriko Toyama, Takehiko Toyosato

**Affiliations:** 1 Department of Mental Health Nursing, School of Health Sciences, Faculty of Medicine, University of the Ryukyus, Ginowan, JPN; 2 Department of Rehabilitation, Ginowan City Community Activity Support Center TAPIC, Ginowan, JPN; 3 Department of Mental Health, Graduate School of Health Sciences, University of the Ryukyus, Ginowan, JPN; 4 Department of Nursing, Faculty of Health Sciences, Yamato University, Suita, JPN; 5 Department of Public Health, School of Health Sciences, Faculty of Medicine, University of the Ryukyus, Ginowan, JPN

**Keywords:** family burden and distress scale, family support, focus group interview, kikuchi's social skills scale consisting of 18 items, schizophrenia, social skills training

## Abstract

Introduction and aim

Although previous psychiatric studies have examined family-based social skills training (SST), limited research has specifically evaluated interventions aimed at improving coping skills among family members of individuals with schizophrenia. This study aimed to evaluate the effectiveness of an SST intervention program for family members of patients with schizophrenia.

Methods

Using a mixed-methods approach, eight family members of schizophrenia outpatients or hospitalized patients receiving treatment at a psychiatric hospital in Okinawa, Japan, were invited to participate. The program consisted of 10 group SSTs conducted once every two weeks from June 2017 to November 2017. Program effectiveness was assessed quantitatively using the Family Burden and Distress Scale (FBDS) and Kikuchi's Scale of Social Skills (KiSS-18), analyzed with the Wilcoxon signed-rank test, and qualitatively via focus group interviews (FGI) conducted before and after the program.

Results

The participants included two fathers, three mothers, and one sibling of the patients (n = 6). Quantitatively, the mean FBDS total score decreased significantly from 60.5 to 51.7 (z = -1.99, p < 0.05), and the mean KiSS-18 score improved significantly from 57.7 to 60.8 (z = -2.20, p < 0.05) after the intervention. Qualitatively, the following three categories were extracted: gained peace of mind by participating in a family meeting; diminished feelings of suffering or burden; and recognition of a change in one's perspective and realization of the effectiveness of coping strategies through actual implementation.

Conclusions

The findings suggest that the SST intervention program may be effective in fostering adaptive coping abilities of family members of patients with schizophrenia. Thus, SST programs organized by medical staff have the potential to ease the emotional burden on families by changing their perception of and coping strategies toward patients. Furthermore, support provided to family members through improvement of their social skills may contribute to an overall improvement in the medical treatment environment for patients and could potentially help reduce the risk of hospital readmission.

## Introduction

The mental health medical welfare system in Japan had long revolved around hospital treatment until 2004, when the Ministry of Health, Labor and Welfare enacted the Health and Welfare for Persons with Disabilities Policy, which encouraged the transition from a hospital-treatment-centered to a community-centered approach. In response to this policy change, guidelines for securing high-quality and appropriate medical care for patients with mental health conditions were established in March 2014, along with the direction of policies toward the community-centered mental health care approach [1]. With this change, the issue has been raised on how to support the community living of individuals with mental illnesses. Since the introduction of these guidelines, establishing a supporting structure for these individuals in the community has been of paramount importance [2].

Close to 90% of patients with mental health conditions live with family members, and it is well known that cohabitation with family members provides the basis for healthy community living for these individuals [3]. In other words, their success in community living is largely contingent on the effort of their family members. Because of this, the burden imposed on the family members of these patients is considerable [4]. Among mental health conditions, schizophrenia tends to impose an especially heavy burden on family members due to its chronic nature. In addition to challenges associated with understanding the illness, responding to various needs of the patient, and financial burden, stigma and prejudice associated with schizophrenia make it difficult for the patient's family to gain understanding or cooperation from the community [5].

Liberman et al. stressed the importance of the patient’s family transitioning into assisting roles in order to improve patient outcomes [6]. Considering this argument, it is especially crucial to evaluate an intervention for families of patients with schizophrenia in enhancing their coping strategies and reducing the burden of care. In addition, the guidelines for care of patients with schizophrenia established by both the National Institute for Health and Care Excellence (NICE) in Britain and the American Psychiatric Association (APA) also highly recommend psychosocial treatment involving the assistance of families. This is because family-assisted interventions for patients with schizophrenia have been shown to be effective in preventing relapse of illness [7,8]. McFarlane et al. advocate the need to expand assistance to families by emphasizing the benefits families bring to patients [9]. Despite the trend toward community living of patients with mental health conditions and the aforementioned potential, details of an effective approach to family-assisted intervention for patients with schizophrenia have not been systematically investigated.

One of the interventions involving families of patients with schizophrenia concerns teaching family members techniques for better managing daily interactions and caregiving challenges. For example, cognitive behavior therapies, focusing on psychoeducation, such as the empowerment assertive communication training program and social skills training (SST), have been applied. In fact, patients with schizophrenia have received SST since the 1970s, and its effectiveness in improving psychosocial functioning is well-established [10]. Moreover, SST has been applied not only to patients with schizophrenia but also to a diverse group, including those suffering from alcohol-related disorder, personality disorder, eating disorder, neurotic disorder, and developmental disorder in a wide range of facilities such as hospitals, daycare centers, halfway houses, and vocational rehabilitation centers. In addition to patients, the patients' families have also received SST [11]. Previous literature on family interventions and SST programs targeting families of patients with schizophrenia has demonstrated structural approaches that are effective in reducing caregiver burden and improving overall family functioning [12,13].

Although previous psychiatric studies have examined family-based social skills training (SST), limited research has specifically evaluated interventions aimed at promoting effective coping behaviors among family members of individuals with schizophrenia. Thus, the purpose of the present pilot study was to obtain information that becomes the foundation for the development of "the family group SST program for family members of patients with schizophrenia" in the future. This was explored through a tentative implementation of a pilot group SST intervention designed to improve family members’ coping strategies for managing schizophrenia-related behavioral and psychological symptoms.

## Materials and methods

Participants

Participants comprised eight family members of outpatients or hospitalized patients with schizophrenia being treated at the psychiatric hospital in Okinawa. Participants were recruited from among family members attending hospital-organized family meetings. Informed consent was obtained from all participants after fully explaining the purpose and details of the study. As a pilot study aiming to evaluate the feasibility and preliminary efficacy of the intervention, the sample size was determined based on the feasibility of recruitment within the study period rather than a formal power analysis.

Summary of the group SST intervention program

Intervention Schedule

The program consisted of one focus group interview, conducted in May 2017 (hereinafter referred to as "focus group interviews {FGI}"), and a total of 10 group SSTs, administered once every two weeks from June to November 2017. After the implementation of group SST, FGI was conducted once to evaluate the effectiveness of the SST intervention.

The Program Implementation

FGI was conducted before the launch of SST. A training agenda for group SST was determined by identifying problems family members experienced in daily life with patients, as well as questions they would like to ask other family members. Group SST, based on these agendas, was implemented 10 times. FGI was conducted again after the implementation of group SST in order to systematically examine the effect of SST intervention on participants' feelings of burden, anxiety, and stress. FGI was specifically chosen over individual interviews to facilitate interaction among participants and capture shared experiences, thereby maximizing the peer-support effect and the richness of the qualitative data.

Program Organization

Table 1 summarizes the agenda and the details for each group SST session. Based on the SST theory, role play was conducted to educate participants on how to respond to a situation for each agenda included in our study program. SST primarily consisted of the following procedures: (1) an information session that provides details about the purpose and the methods of SST, (2) ice-breaker activities including self-introduction, (3) role playing, and (4) a group discussion. While focusing on their improvement, participants were encouraged to commend each other on their good deeds. This effort was intended to facilitate behavior change through the power of positive thinking. Each session lasted for 50-60 min.

**Table 1:** The program agendas and specific coping strategies. SST: social skills training

Session	Agenda	Details	Specific examples of coping
The first SST	Excessive drinking of water.	Drink 5-6 L of water per day.	Ask the patient why he/she drinks excessive amounts of water. Lovingly praise the patient when he/she is able to withhold drinking water. Changing the tone of voice when talking to a child, focus on being gentle when talking to the patient. In order for the patient to be aware of their own weight gain, get the patient involved in confirming their weight measurement.
The second SST	Timing of saying something to the patient.	How to respond when the patient yells or how to talk to the patient when their condition is not good, or they are in a bad mood.	First, the parents must deepen their understanding of their child’s condition or illness and respond accordingly. Increase opportunities for communication, such as greeting in the morning. Respond to the child while reminding ourselves that avoiding conflict with the child is the key. Identify ways, such as topics the patient likes or small talk techniques, to strike up a conversation with the patient.
The third SST	Collecting items.	How to respond when the patient buys things that are clearly unnecessary.	Ask why the patient bought what he/she bought. Ask the patient to keep a record of revenues and expenditures. Learn financial management with the assistance of a third party, such as home health nurses.
The fourth SST	From the standpoint of the patient’s sibling.	I am concerned about how the government policies and the system of medical services in Japan will change hereafter.	In addition to medical care, lead daily lives with the assistance of a helper. Take advantage of social work- or nursing-care services. Share the hardship each family went through when responding to the problematic behaviors of the patient.
The fifth SST	Wanting to have a conversation with my son, but unsure how.	I don’t receive a response even when I greet the patient.	Guide the patient’s response by talking about the weather or food. Do the housework together with the patient. Praise anything that the patient was able to do.
The sixth SST	How to respond to abusive words, as well as the management of finances.	When the topic of assets comes up, the patient uses abusive words.	Avoid talking in whispers or forcing the patient’s attention by persistently talking to the patient. In terms of asset and property management, the parents’ will should be preserved in documents for the future. Consult the medical staff at the hospital when the patient is in an unfavorable condition.
The seventh SST	Responding to obsessive behavior.	The patient constantly washes their hands.	Ask the patient about the reasons for washing their hands. Praise the patient for their cleanliness. Talk to the patient in a kind and gentle tone. Let the patient know that everyone in the family is worried. Avoid denying whatever the patient is doing.
The eighth SST	Symptoms of violence.	Because the patient refused to take medication, he/she experienced hallucinations.	We would like the medical staff to inform the family about the illness. The family would like to be heard.
The ninth SST	Not being able to accept anything other than what the patient decided.	The patient constantly cleans a certain section of the house.	Acknowledge the way the patient is handling things, show respect to the patient’s efforts, and praise the patient. Have the patience to wait and watch the patient silently. Do not deny the way the patient is handling things. Gain the cooperation of the third party.
The 10th SST	Timing concerning when to move into a group home or live alone.	I want the patient to become self-reliant.	Ask the hospital if it is possible for the patient to live alone. Ask the patient whether they are willing to live by themselves. Tell the patient that their parents will not be well enough to take care of the patient forever. Kindly talk to the patient about the possibilities of tiered independent living by saying, “He/she should start practicing living on their own.”

Evaluation index

In order to assess the effectiveness of the group SST intervention program, an evaluation was performed before and after the group SST intervention with a questionnaire and FGI. Questionnaires were distributed and collected in person at the hospital immediately before the first session and after the final session. To ensure privacy and anonymity, participants were instructed to respond anonymously.

Family Burden and Distress

The Family Burden and Distress Scale (FBDS) was selected to identify perceived challenges or feelings of burden felt by the family towards the onset of schizophrenia in the patient. This scale has been verified by Yamaguchi et al. for internal consistency, reproducibility, criterion-related validity, and construct validity [14]. Permission to use this scale was obtained from the developer. One of the most salient advantages of FBDS lies in its ability to grasp various psychological phenomena and challenges regarding the patient's family, including the shock associated with the diagnosis, difficulties arising from the lack of knowledge or information about the illness, anxiety about the future, depression, feelings of loneliness, and other issues surrounding their relationship with the patient.

FBDS is comprised of 24 items assigned to the following three categories: (1) perceived burden or depression arising from the patient's illness (hereinafter referred to as "perceived burden or depression"), (2) confusion arising from lack of knowledge concerning illness and anxiety about the future (hereinafter referred to as "confusion due to lack of knowledge and anxiety about the future"), and difficulty concerning their relationship with the patient (hereinafter referred to as "relationship difficulty"). The perceived burden or depression category consists of 10 items; some examples are as follows: "I feel physical exhaustion," "I became unable to maintain open and honest communication with friends and relatives," and "I cannot sleep well." The confusion due to lack of knowledge and anxiety about the future category consists of nine items; some examples are as follows: "I don't know what I make of the future," "I cannot understand the patient's behavior," and "I have no idea when the current issues will resolve." The relationship difficulty category consists of five items; some examples are as follows: "I tend to overreact to the patient," "I often talk to the patient," and "I feel irritated when being with the patient." Participants were asked to indicate their perceived challenges and feelings of burden with a four-point Likert scale ranging from 1 (completely true) to 4 (not at all true) on the FBDS. The higher score indicates greater subjective challenges and feelings of burden [14].

Social Skills

Kikuchi's Social Skill Scale, consisting of 18 items (hereinafter referred to as "KiSS-18"), was used to assess the ability of family members to cope with the patients. The reliability and validity of KiSS-18 have been verified in a wide range of populations, from adults to the elderly, in a number of studies conducted in Japan [15]. Permission to use this scale for academic research was confirmed by the publisher. This scale is also publicly available for non-commercial academic research. KiSS-18 rates family members in the following six areas: basic skills, high-level skills, emotion-processing skills, anger-management skills, stress-coping skills, and planning skills. The participants were asked to indicate the most appropriate answer from 1 (never the case) to 5 (always the case) on a five-point Likert scale. A total score was calculated for each participant. The higher the KiSS-18 score, the more sophisticated the participant's social skills.

Data analyses

Due to the small number of participants in this study, the effect of the SST group intervention was assessed with a non-parametric test. The Wilcoxon signed-rank test was used to assess whether the difference before and after the intervention is statistically significant. All statistical analyses were conducted using SPSS Statistics version 22.0 (Armonk, NY: IBM Corp.).

Also, the effectiveness of the SST intervention program was assessed with the qualitative inductive analysis. Qualitative analysis was essential to understand the subjective process of participants' behavioral changes that quantitative scales alone might not fully capture. We recorded FGI with a digital voice recorder after the implementation of SST. Statements reflecting changes in participants’ perceptions, attitudes, and coping experiences were extracted from the interview transcripts. Based on the similarity, the records were first classified into a subcategory and then into a category. During analysis, our team regularly referred to the verbatim transcription to determine whether grouping of information into certain categories is appropriate.

## Results

Participants

Initially, eight family members of patients with schizophrenia consented to participate. However, a compliance rate of 75% or higher (attending at least eight out of 10 sessions) was required for the final analysis to ensure sufficient exposure to the intervention efficacy. Therefore, six participants, comprising two fathers, three mothers, and one sibling of the patients (n = 6), were included in the final analysis. The two participants who were unable to attend the required number of sessions were excluded. Regarding other sociodemographic and clinical characteristics, three participants were in their 60s, two in their 70s, and one in their 80s, with an average age of 71 years. Their educational backgrounds included university graduates (n = 3), high school graduates (n = 2), and a vocational school graduate (n = 1). In terms of financial status, five participants reported their economic situation as "somewhat comfortable," and one reported it as "comfortable." Additionally, two of the six participants had chronic medical conditions as follows: one had hypertension and gout, and the other had asthma and hypertension.

Change in FBDS and KiSS-18 scores before and after the intervention

Among the six participants, three improved their scores on the FBDS and KiSS-18 (Table 2). Two participants also improved their scores on the FBDS and KiSS-18, except for one domain (i.e., relationship difficulty) of the FBDS. Quantitatively, the mean FBDS total score decreased significantly from 60.5 to 51.7 (z = -1.99, p < 0.05), and the mean KiSS-18 score improved significantly from 57.7 to 60.8 (z = -2.20, p < 0.05) after the intervention, indicating statistically significant improvements in both perceived burden and social skills (Table 3).

**Table 2:** Change in FBDS and KiSS-18 scores of each participant (n = 6). *Subjective burden and depression resulting from the patient's illness. **Confusion resulting from a lack of knowledge of illness and anxiety. ***Difficulties in the relationship with the patient. KiSS-18: Kikuchi's Scale of Social Skills; FBDS: Family Burden and Distress Scale

Participant ID	FBDS total score		Subjective burden*		Confusion**		Difficulties***		KiSS-18	
	Pre	Post	Pre	Post	Pre	Post	Pre	Post	Pre	Post
1	67	68	21	24	32	30	14	14	52	52
2	59	53	20	19	31	24	8	10	54	57
3	53	47	21	20	21	19	11	8	65	70
4	45	29	11	10	27	13	7	6	70	71
5	67	52	32	22	22	17	13	13	62	64
6	72	61	24	20	33	28	15	13	43	51

**Table 3:** Evaluation and change of mean and median in FBDS and KiSS-18 before and after intervention (n = 6). *Subjective burden and depression resulting from the patient's illness. **Confusion resulting from a lack of knowledge of illness and anxiety. ***Difficulties in the relationship with the patient. FBDS: Family Burden and Distress Scale; KiSS-18: Kikuchi's Scale of Social Skills

Variables	Pre				Post				z-Value	p-Value
	Mean	SD	Median	Range	Mean	SD	Median	Range		
FBDS total score	60.5	10.2	63.0	45-72	51.7	13.4	52.5	29-68	-1.99	0.046
Subjective burden*	21.5	6.8	21.0	11-32	19.2	4.8	20.0	10-24	-1.36	0.168
Confusion**	27.7	5.2	29.0	21-33	21.8	6.6	21.5	13-30	-2.20	0.027
Difficulties***	11.3	3.3	12.0	7-15	10.7	3.2	11.5	6-14	-1.68	0.357
KiSS-18	57.7	9.9	58.0	43-70	60.8	8.8	60.5	51-71	-2.20	0.043

FGI results

Table 4 summarizes the results of the FGI analysis following the intervention. The following three primary categories were identified: "gained peace of mind by participating in a family meeting," "diminished feelings of suffering or burden and recognition of the change in one's perspective," and "realization of effectiveness in coping strategies through actual implementation."

**Table 4:** A focus group interview conducted after participating in SST. SST: social skills training

Categories	Subcategories	Narratives
Participating in a family meeting gave me a sense of security and relief.	Understanding the illness as a series of symptoms.	I remember referring to my child as lazy, but I no longer think of him/her like that. Now, I can see his/her tendency as a part of his/her condition due to the illness.
		I was able to embrace my child’s condition as a part of their symptoms.
	Gaining relief by expressing my agony (spitting out the agonies).	I cannot talk about my child at the elderly society/club.
		I cannot help but flash back into the traumatic experiences of the past. My feelings just go back to the past somehow. Maybe all the hardships occurred in the past.
		Doctors tell us that we can recover from a traumatic experience by talking about it, but that does not quite happen for me. Every time I try talking about the traumatic experience, the memories come flashing back.
		I ponder over many issues alone, not being able to tell anyone.
	I was able to heal and gained a sense of relief and peace of mind.	I am so, so glad I participated in the family meeting. It is a really great opportunity. The family meeting truly healed me.
		I was able to gain a sense of peace by sharing common ground with a group of people going through similar hardships.
		I consider the opportunity of meeting other families itself as a valuable asset. Please organize the family meetings at the hospital, too. I would like to make a request for that in this kind of friendly atmosphere.
		I can feel that I, too, was becoming ill. I was able to be healed by interacting with others in the family meeting. After all, I was largely being helped by the grace and kind souls of others.
		I was truly able to gain some peace of mind and really felt relieved.
		Every family deals with different symptoms, and every child has distinct symptoms. Regardless of the differences in the symptoms we are dealing with, everyone is trying their hardest. Just hearing what others are going through gives me a sense of support and the courage to keep going.
The feeling of agony or burden has been alleviated, and I recognized the change in my perception.	The sense of burden has been alleviated.	I realized that I was not alone.
		I realized there are so many things that others are able to do.
	Recognized the change in myself.	I no longer suffer from the nightmares that haunted me prior to May, when this meeting started. I no longer quarrel with my spouse, and am noticing that I am gaining a sense of peace again. I am noticing the change.
		The best thing might be, if we change, our child might change. I can feel that I have changed. I feel it now.
		I have gone through a major transformation since I participated in this meeting. I became able to control fluctuations in my emotions.
		I think it is important to take the time to care for my child as well as the time to do other things. Otherwise, I will not be able to hold myself up.
		My child’s emotions are not stable, even in good times, and obviously in bad times. I can easily care for my child in good times, but in bad times, it becomes impossible. So, I am constantly tense under the nerve-wracking, stinging pressure.
		I realized I was overthinking. It gradually became clear to me that I have been becoming obsessed with caring for my child.
	The meeting helped me to develop the emotional capacity needed to respond to various challenges.	I became able to secure time for myself by joining a circle or an elderly society/club.
		I became able to secure my time for hobbies.
I was keenly able to feel the effectiveness of coping strategies and skills after actually implementing them hands-on.	It became easier to talk to my child.	I am glad I was able to learn how to talk to or to start a conversation with my child.
		It is easier to talk to my child than before.
	I was able to keenly feel the effectiveness of coping strategies after implementing them.	I became able to change my coping strategies for the better after what I learned in SST.
		I was able to receive feedback from the group about my son’s excessive consumption of water; since then, it has subsided.
		I no longer fight or quarrel with my son or with my spouse.
	I gained a deeper understanding of my child’s circumstances and the illness.	I wonder what the next step will be. I wonder if we will be able to receive coordinated medical care assistance at the care facility.

The category "gained peace of mind by participating in a family meeting" was derived from the following three subcategories: "understanding an illness from symptoms," "expression of agony," and "feeling of comfort through self-healing." The second category, "diminished feelings of suffering or burden and recognition of the change in one's perspective," comprised "ease of burden," "recognizing the change in oneself," and "gaining emotional leeway." Finally, the category "realization of effectiveness in coping strategies through actual implementation" emerged from the subcategories "having an easier time talking to the patient," "realizing the effectiveness of training through actual implementation," and "gaining a deeper understanding of the patient's condition or illness."

## Discussion

The present study has several limitations. First, this was a pilot study aimed at evaluating the feasibility and potential effect of the SST group intervention. Because the final analysis was limited to a small sample size (n = 6) and lacked a control group, statistical power is limited, and caution must be exercised when generalizing the results to a larger group of families; therefore, further controlled studies with a greater sample size are warranted. Furthermore, two of the initial eight participants were unable to complete the program due to health issues and work scheduling conflicts. This highlights the practical challenges in maintaining long-term attendance in family interventions. Second, there has been a delay in publication since the data collection in 2017, primarily due to the time required for a meticulous re-analysis of the mixed-methods data. However, the clinical significance of this program remains highly relevant today. Since the 2022 revision of Japan's Act on providing comprehensive support for the daily life and life in society of persons with disabilities (Act No. 104 of 2022, the policy emphasis on family support and community-based mental health care has accelerated [16]. Because structured, evidence-based family SST programs remain extremely scarce in Japanese clinical settings, these foundational data continue to be crucial. Despite these limitations, the present study is valuable in that it identified the potential of group SST interventions for families of patients with schizophrenia. Future studies should build on this potential and evaluate other intervention techniques and activities besides role play, such as reflection, active listening, clarification, and summarizing. Furthermore, to enhance the sustainability and effectiveness of family interventions, future research should integrate variables such as sleep cycles (circadian rhythms), digital device usage patterns, sociocultural practices, and the potential introduction of early mental health biomarkers.

The purpose of the present study was to develop and evaluate the effectiveness of the group SST program for the families of patients with schizophrenia. After the group SST intervention, the reported feelings of suffering and burden among family members had been alleviated, and self-reported scores of social skills had improved. Therefore, it is reasonable to conclude that the group SST intervention has the potential to provide effective improvement in families of patients with schizophrenia. The comparison before and after the intervention showed how effective the group SST was in easing perceived feelings of challenge or burden among family members. Through the opportunity of attending the group SST meetings, the family members of patients with schizophrenia were able to learn more appropriate coping strategies from other families. Although the feeling of anxiety and agony was tremendous among family members when responding to challenging situations patients faced, this feeling seemed to have transformed into a more positive attitude after the intervention. These findings indicate that the intervention may have reduced psychological distress among participating family members.

Results of the qualitative analyses suggest how sharing pent-up feelings of agony with other family members in a safe environment led to an overall positive experience as summarized in narratives such as "I became able to see schizophrenia simply as a series of symptoms," "I used to suffer alone, not being able to tell anyone how I felt, but it is different now," "participating in SST group facilitated emotional healing," and "having supportive people going through similar experiences is an asset." The fact that some participants reported, "I became able to secure the time for myself," might be evidence that gaining emotional leeway by joining the SST group contributed to the eased feeling of burden or obligation on the family's part. Yalom proposed that one of the factors contributing to a curative effect in group therapy arises from the sense of security that emanates from having hope and being a part of the community [17]. Interaction with peers facing similar caregiving challenges appeared to promote mutual understanding, emotional validation, and shared learning [18]. We were able to promote the feeling of empathy and sense of security among family members who participated in the SST group by emphasizing the fact that difficult problems occur in all families and each family is not suffering alone. This seems to have contributed to making SST group meetings a more comfortable place for all participants. Furthermore, the intervention was effectively able to improve the social skills of participants.

After identifying the needs of families with FGI, we employed a role-play technique to help participants learn how to respond appropriately to each difficult situation. Families described solid, positive outcomes of the intervention in the narratives used for qualitative analyses as follows: "I was happy to learn how to talk to and strike up a conversation with the patient," "talking to the patient became easier than before," and "I am less likely to fight with the patient." We presume that the approach we employed in the group SST has improved the social skills of family members in the two aspects. Firstly, the role-play technique allowed simulation of difficult situations, thereby helping them learn how to appropriately respond to a specific situation in a real-life setting. Secondly, it is likely that sharing their plights as well as successes with other families provided meaning to their struggles because, by sharing their experience with other families, they realized they were able to help other families that are going through similar experiences. This may have transcended the extremely negative experiences and created positive feelings in those families.

## Conclusions

The findings suggest that these changes in the perception of families may have greatly contributed to the improvement in their social skills. Based on these considerations, the present study highlights the potential of intervention programs organized by medical staff for family members of patients with schizophrenia to ease their emotional burden by changing their perception of and coping strategies toward the patient. Furthermore, support provided to family members through the improvement of their social skills may contribute to an overall improvement in the medical treatment environment for the patients, which could potentially help reduce the risk of hospital readmission.
